# Targeted nanopore sequencing for the identification of novel *PRMT1* circRNAs unveils a diverse transcriptional profile of this gene in breast cancer cells

**DOI:** 10.1016/j.gendis.2023.04.013

**Published:** 2023-05-18

**Authors:** Maria Papatsirou, Andreas Scorilas, Diamantis C. Sideris, Christos K. Kontos

**Affiliations:** Department of Biochemistry and Molecular Biology, National and Kapodistrian University of Athens, Panepistimiopolis, Athens GR 15701, Greece

Long-read sequencing with nanopore technology has been widely utilized for the identification of the full-length sequence of RNA molecules.^1^ It is especially suitable for the identification of circular RNAs (circRNAs), which are regulatory molecules that are regarded as promising non-invasive biomarkers for various human malignancies, including breast cancer.^2^ The impact of circRNAs derived from the protein arginine methyltransferase 1 (*PRMT1*) gene has not been clarified yet, even though the effect of PRMT1-mediated methylation on breast cancer cell characteristics has been well established. We have previously shown that several circRNAs are produced by *PRMT1*,^3^ and thus we aimed at identifying novel *PRMT1* circRNAs in 12 breast cell lines, following a PCR-based, targeted nanopore sequencing workflow (described in detail in the supplementary materials and methods). This pipeline leads to the identification of the full circRNA sequence, including the back–splice junction, as a single read. Contrary to circRNA profiling studies, our targeted approach allows for the amplification and sequencing of circRNAs originating from the *PRMT1* gene with higher specificity. Meanwhile, it enables the identification of rare circular transcripts that may be missed by a direct circRNA sequencing approach. In our study, a wide variety of novel *PRMT1* circRNAs were identified. Their intricacy in structure and distinct expression profiles among breast cancer cells raises new questions about the functions of circRNAs and the regulatory role of *PRMT1* in breast cancer.

In this study, 11 breast cancer cell lines of distinct molecular subtypes (Table S1) and a normal breast cell line (MCF-12A) were used. Following cell lysis and total RNA extraction from each cell line, reverse transcription was carried out using random hexamers. In the following PCR assays, we used two divergent primer pairs complementary to each exon of the *PRMT1* gene (Table S2). Thus, the amplification of circRNAs comprising exclusively intronic areas of this gene was ruled out. Equal volumes of the nested PCR products from the cDNA of each cell line were mixed, prior to adapter ligation and sample barcoding used for DNA library construction. Targeted third-generation sequencing based on the nanopore technology was performed in a MinION Mk1C device carrying the Flongle adapter (Oxford Nanopore Technologies plc.). To process our data, we used several algorithms, including Minimap2, SAMtools, BEDtools, ASDT, ASDT remodeler, and Read catcher, as illustrated in Figure S1. The selected reads representing circRNAs were then manually annotated. Finally, the prediction of the miRNA-binding sites of the novel circRNAs comprising genomic regions beyond the known boundaries of the *PRMT1* gene was performed with the custom prediction tool of the miRDB database.

By the completion of the sequencing run, 374,000 reads had been generated, with an approximately equal read number between the 12 breast cell lines (Fig. S2A, B). The percentage of aligned reads to the *PRMT1* genomic region for each of the 12 cell lines ranged from 84.3% to 91.7% (Fig. S2C). The mean length of reads produced from the sequencing experiment was around 400 nucleotides (nt) for all cell lines (Table S3). Following our sequencing data analysis workflow, 120 *PRMT1* circRNAs comprising a single exon or multiple exons, were finally annotated. Out of these, 1 *PRMT1* circRNA had previously been deposited in circAtlas (circ-PRMT1-36) and 4 circRNAs had already been deposited in circBase (circ-PRMT1-23, circ-PRMT1-36, circ-PRMT1-91, and circ-PRMT1-100). Most novel circRNAs consist of up to 5 *PRMT1* exons, while their length varies between 153 and 1627 nt (Fig. S2D, E). Through the comparison of the *PRMT1* circRNA expression profile between the cell lines, prevalent differences arise, such as the frequency of intron retention and exon extensions, as well as the existence of novel *PRMT1* exons (Fig. 1A). However, there seems to be no obvious correlation between the molecular subtype of breast cancer and the circRNA expression pattern (Fig. S3), even though the expression analysis of the identified circRNAs revealed several circRNAs showing molecular subtype-specific expression (Table S4). Moreover, we performed copy number variation (CNV) analysis for *PRMT1* in the 11 breast cancer cell lines, based on the Cancer Cell Line Encyclopedia, in order to assess any correlation with the number of identified *PRMT1* circRNAs. Although 7 cell lines have copy number gains of this gene and 4 have a diploid copy of it, no obvious correlation was observed between the CNV of *PRMT1* and the novel circRNAs that are expressed in these cell lines (Fig. S2F). Interestingly, 6 circRNAs were found only in the non-cancerous breast cell line (Fig. 1B). These differences in the expression profile of *PRMT1* circRNAs point towards a strictly regulated biogenesis mechanism under specific conditions. Moreover, it is obvious that several genomic regions that are encountered in the novel *PRMT1* circRNAs are not present in any of the annotated linear transcripts of this gene (Fig. 1A).

**Figure 1:**
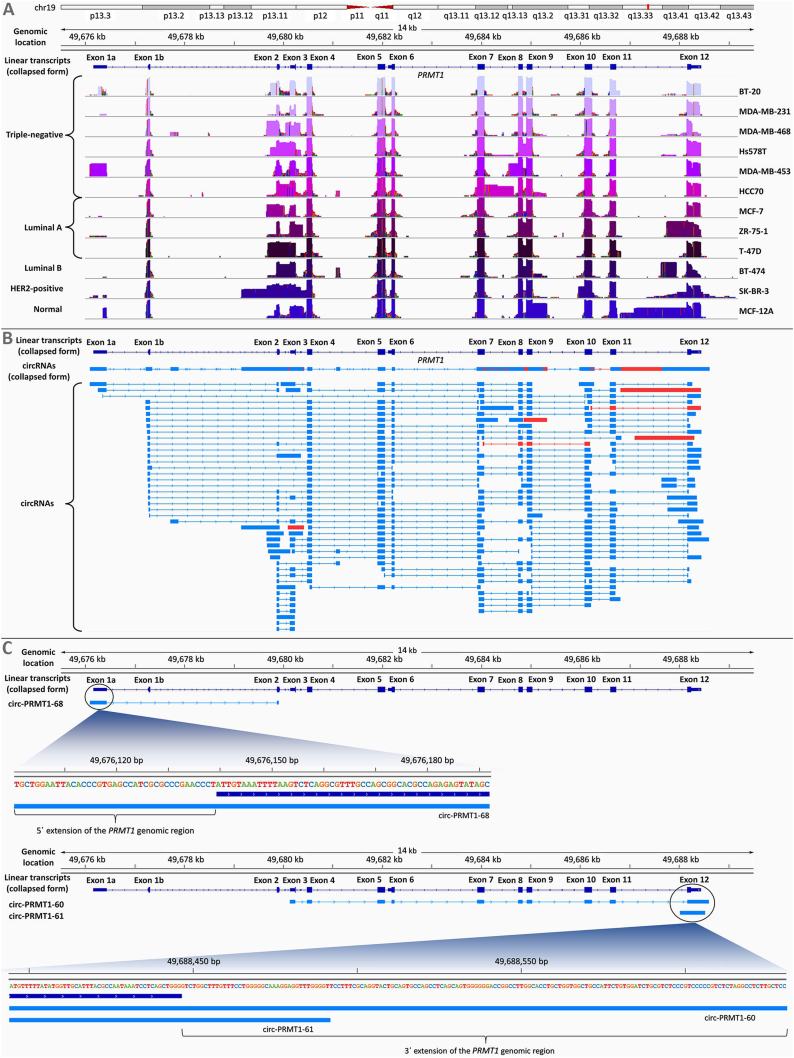
Nanopore sequencing results evidence the wide variety of circRNAs transcribed by *PRMT1*, including circRNAs comprising sequences beyond the currently known gene boundaries. **(A)** Coverage of the *PRMT1* gene by the circRNAs expressed in each one of the 12 cell lines used in this study, using the Integrative Genomics Viewer (IGV); the illustration of the aligned reads of nanopore sequencing is presented in logarithmic scale. The cell lines are grouped based on the breast cancer molecular subtype. Distinct expression patterns of *PRMT1* circRNAs are observed in the cell lines. **(B)** Alignment of the newly identified *PRMT1* circRNAs against the genomic sequence of *PRMT1*. The previously annotated, linear *PRMT1* transcripts are shown in a collapsed form (dark blue color); below, the novel circular transcripts of this gene are shown in collapsed and expanded form. Novel circRNAs that were identified only in the MCF-12A cell line are shown in red color. **(C)** Alignment of the 3 *PRMT1* circRNAs that include 5′- or 3′-extensions of the currently known *PRMT1* genomic region. The first aligned exon of circ-PRMT1-68 starts before the already known transcription start site of the *PRMT1* gene, having a 5′-extended region of 39 nucleotides (nt). The last aligned exon of circ-PRMT1-60 ends after the currently known poly(A) site of the *PRMT1* gene, similar to the single-exon circ-PRMT1-61. These circRNAs contain 3′-extended regions of the gene (159 nt and 39 nt, respectively).

From the annotation of the sequencing reads, it was evident that most back–splice junctions were characterized by non-canonical alternative splice sites and truncated *PRMT1* exons, usually resulting in microexons being back-spliced. Recent research suggests that microexons are preferentially found in circRNAs,^4^ and this provides a notion that circRNA-specific RNA elements could have unique functions that cannot be attributed to the linear transcripts. Moreover, non-canonical splicing events are prevalent in cancer; they disrupt transcriptome integrity by evading cellular quality control systems.^5^ Therefore, some novel *PRMT1* circRNAs that incorporate non-canonical splice sites are likely to disrupt regular gene expression and alter the ratio of normal to pathological transcript variants in a cancer setting. Additionally, 3 novel exons of the *PRMT1* gene were discovered and shown to participate in circRNAs (Fig. S4A). Interestingly, novel exons #1 and #3 participate in the back–splice junction of the circRNAs they are incorporated in (Fig. S4B). None of the 3 novel exons were previously discovered in linear *PRMT1* transcripts through sequencing experiments; thus, it could be hypothesized that these exons are exclusively present in circRNAs generated by the *PRMT1* gene. circRNA-specific exons may bind splicing components or introduce RNA structures that preferentially promote back-splicing than forward-splicing junction formation. Another reason for the fact that these exons have not been detected in *PRMT1* mRNAs so far could be that linear transcripts bearing them are likely to be subjected to nonsense-mediated decay in the cytoplasm, due to premature translation termination codons generated by frameshifting. Interestingly, using the ORF Finder bioinformatics tool, we found that all circRNAs incorporating novel *PRMT1* exons possess an open reading frame *in silico*. Therefore, if translated, these novel PRMT1 protein isoforms would contain additional internal peptides, probably affecting the tertiary structure of the resulting polypeptides.

Another feature observed in all cell lines was the presence of 5′- and/or 3′-extensions of the previously annotated *PRMT1* exons. The novel exon ends participate in newly discovered back–splice junctions. More interestingly, the MCF-12A cell line, which originates from normal breast epithelium, characteristically differs from the breast cancer cell lines regarding the exon extensions that are incorporated in its *PRMT1* circRNAs. The extended exons found in some of these *PRMT1* circRNAs contain sequences that are absent from the linear *PRMT1* transcripts, thus suggesting binding partners probably distinct from those of the *PRMT1* mRNAs. The presence of additional binding sites for miRNAs or proteins could result in alterations in the stability and/or localization of such circRNAs. Moreover, this finding may indicate yet unknown features of the back-splicing mechanism(s). In several cell lines, full intron retention was observed as well, in contrast with *PRMT1* mRNAs.

Another unexpected finding was that some *PRMT1* circRNAs comprised regions residing outside the boundaries of the *PRMT1* gene. More specifically, a circRNA (circ-PRMT1-68) with a 5′-extension of the predicted first *PRMT1* exon (exon 1a) is expressed in the MDA-MB-453 cell line, while two circRNAs (PRMT1-60 and circ-PRMT1-61) with distinct 3′-extensions of exon 12 were found in the SK-BR-3 cell line (Fig. 1C). These findings redefine the boundaries of the *PRMT1* gene and suggest the existence of longer untranslated regions even in the mRNAs produced by this gene. The existence of miRNA-binding sites in the sequences of these regions was queried; in fact, putative binding sites for miR-4284 and miR-24-3p appear in circ-PRMT1-68, whereas a binding site for miR-361-3p is predicted for both circ-PRMT1-60 and circ-PRMT1-61. These three miRNAs exert an oncogenic role in breast cancer; thus, by sponging these miRNAs, these three *PRMT1* circRNAs might play a key role in modulating breast cancer cell survival. In addition, despite circRNAs being generally considered RNA molecules that lack a poly(A) tail, we discovered 2 circRNAs that contain multiple consecutive adenosine monophosphates next to the nucleotide that constitutes a poly(A) site in linear *PRMT1* transcripts (Fig. S4C). The end of both poly(A) stretches participates in the back–splice junction of the respective circRNAs, thus raising questions regarding the order of maturation events of the primary *PRMT1* transcripts leading to circRNA biogenesis and the splicing factors involved in this back-splicing mechanism.

In conclusion, it is evident that *PRMT1* is a gene producing a variety of circRNAs that encompass multiple intricate alternative splicing events. The transcriptional profile of this gene differs between molecular subtypes of breast cancer, and between breast cancer cell lines as well. Therefore, these circRNAs are expected to possess distinct molecular features and functions. For instance, several *PRMT1* circRNAs contain putative ORFs, including those containing novel exons or poly(A) stretches. Their translation could be dependent on m^6^A modifications that are usually present in circRNAs. However, RT-PCR-based workflows have an inherent limitation as they can sometimes result in artifacts, for instance by template switching of the MMLV reverse transcriptase. For this reason, the expression levels of the novel *PRMT1* circRNAs as well as their secondary structure and cellular topology are aspects necessitating future investigation, to elucidate their role in breast cancer.

## Author contributions

Maria Papatsirou performed experiments, collected and analyzed data, drafted the manuscript, and produced figures. Andreas Scorilas provided resources and critically reviewed the manuscript. Diamantis C. Sideris critically reviewed the manuscript. Christos K. Kontos conceived the study, designed experiments, had supervision, and critically reviewed the manuscript. All authors read and approved the final version of this manuscript.

## Conflict of interests

The authors have no conflict of interests to declare.

## Figures and Tables

**figs1:**
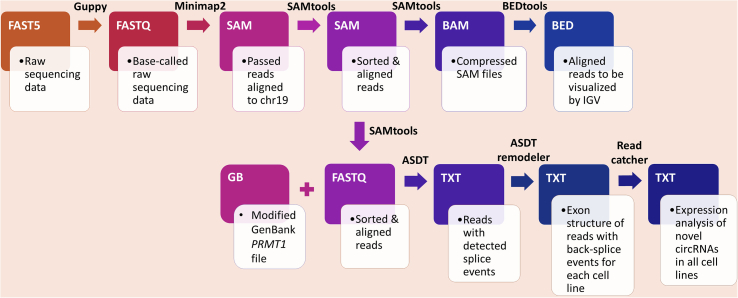
**Supplementary Figure 1.** The step-by-step bioinformatics pipeline that was followed in this study. The nanopore sequencing data analysis started from the FASTQ files that were generated by the MinKNOW software; the reads were aligned, sorted, indexed, and visualized with the Integrative Genomics Viewer (IGV) or further analyzed with PERL-based algorithms, to identify novel *PRMT1* circRNAs through manual annotation.

**figs2:**
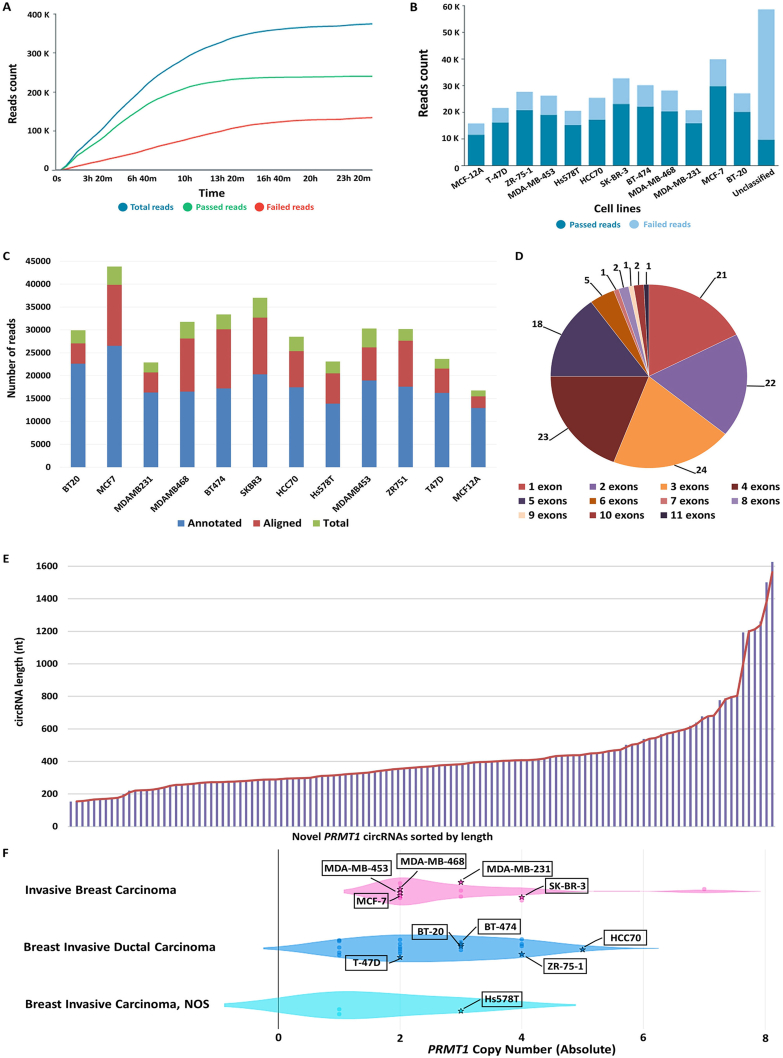
**Supplementary Figure 2.** Metrics from the nanopore sequencing experiment and the annotation of the results. **(A)** Chart depiction of the total, passed, and failed reads that were generated during the 24 hours of sequencing run. **(B)** Bar plots depicting the distribution of passed and failed reads for each cell line. In cases where the barcode could not be categorized, the reads were tagged as “Unclassified” and were not used in downstream analysis. **(C)** The distribution of total passed reads, reads aligned to the *PRMT1* gene locus, and annotated reads for each cell line. For all 12 cell lines, more than 85% of passed reads were aligned to the *PRMT1* gene, and an average of 80% (range: 64%-94%) of aligned reads correspond to annotated novel *PRMT1* circRNAs that were identified in this study. **(D)** Depiction of the number of *PRMT1* exons that are incorporated into the identified circRNAs of the gene. The majority of the novel *PRMT1* circRNAs consist of 1 to 5 exons. **(E)** The distribution of the length of the novel *PRMT1* circRNAs. The majority of circRNAs have a length between 200 nt and 600 nt. The moving average trendline is also depicted, in order to smooth out fluctuations in the values. **(F)**Frequency plot of the copy number variation of *PRMT1* in the 11 breast cancer cell lines grouped by disease subtype, according to the DeepMAP portal of the Cancer Cell Line Encyclopedia (CCLE). Out of the 11 cell lines, 4 have a diploid copy number of *PRMT1*, while the other 7 are characterized by 1-3 copy number gains of the gene.

**figs3:**
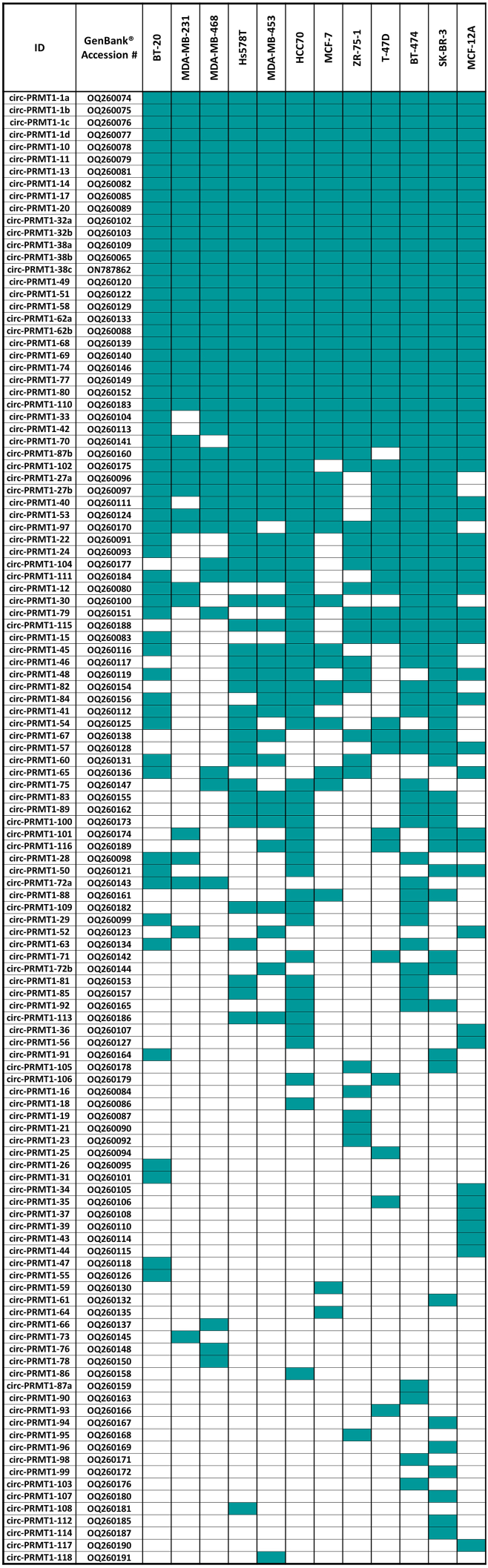
**Supplementary Figure 3.** Illustration of the presence of all identified novel *PRMT1* circRNAs across the 12 cell lines, sorted by frequency. The teal blue color indicates the presence of the respective circRNA in a cell line, according to the results of our targeted nanopore sequencing workflow. For each circRNA, the GenBank® accession number is shown as well.

**figs4:**
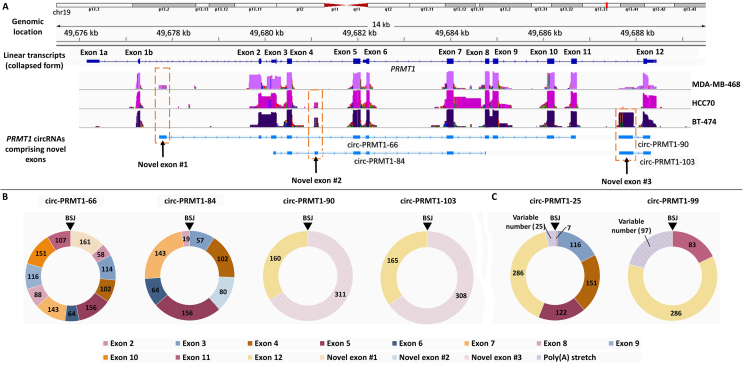
**Supplementary Figure 4.** Representation of the 3 novel *PRMT1* exons, in linear and circular form, as well as the circRNAs that contain poly(A) stretches. **(A)** Depiction of the coverage of the *PRMT1* gene in the 3 breast cancer cell lines where the novel exons were found (logarithmic scale), as well as the alignment of the circRNAs that contain these novel exons, through the Integrative Genomics Viewer. Notably, novel exon #2 is found to be expressed in both the HCC70 and BT-474 cell lines, while novel exon# 3 is incorporated into 2 circRNAs and is the most frequent novel exon. **(B)** The exonic composition of the *PRMT1* circRNAs that contain the 3 novel exons, shown in circular form (not on scale). These circRNAs vary in their length as well as their structure, and novel exons #1 and #3 participate in the back-slice junction (BSJ). Regarding novel exon #3, it was found with 2 different 3΄ splice sites; in circ-PRMT1-90 it is spliced with the known 5΄ end of exon 12, while in circ-PRMT1-103 it is 3΄ truncated and spliced with a 5 nucleotide 5΄ extension of exon 12. **(C)** The exonic composition of the *PRMT1* circRNAs that incorporate poly(A) stretches, shown in circular form (not on scale). These circRNAs were found in the T-47D and SK-BR-3 cell lines, respectively. In both cases, the poly(A) stretches participate in the back-splice junction (BSJ).
