# Supplementary material for: Targeted nanopore sequencing for the identification of novel PRMT1 circRNAs unveils a diverse transcriptional profile of this gene in breast cancer cells
Source: Genes Dis. 2023 May 18;11(2):589–92. doi: 10.1016/j.gendis.2023.04.013 (PMC10491911; doi:10.1016/j.gendis.2023.04.013)
Supplement: Multimedia component 1 [file mmc1.docx]

**Supplementary Figure Legends**

**Supplementary Figure 1.** The step-by-step bioinformatics pipeline that was followed in this study. The nanopore sequencing data analysis started from the FASTQ files that were generated by the MinKNOW software; the reads were aligned, sorted, indexed, and visualized with the Integrative Genomics Viewer (IGV) or further analyzed with PERL-based algorithms, to identify novel *PRMT1* circRNAs through manual annotation.

**Supplementary Figure 2.** Metrics from the nanopore sequencing experiment and the annotation of the results. **(A)** Chart depiction of the total, passed, and failed reads that were generated during the 24 hours of sequencing run. **(B)** Bar plots depicting the distribution of passed and failed reads for each cell line. In cases where the barcode could not be categorized, the reads were tagged as “Unclassified” and were not used in downstream analysis. **(C)** The distribution of total passed reads, reads aligned to the *PRMT1* gene locus, and annotated reads for each cell line. For all 12 cell lines, more that 85% of passed reads were aligned to the *PRMT1* gene, and an average of 80% (range: 64%-94%) of aligned reads correspond to annotated novel *PRMT1* circRNAs that were identified in this study. **(D)** Depiction of the number of *PRMT1* exons that are incorporated into the identified circRNAs of the gene. The majority of the novel *PRMT1* circRNAs consist of 1 to 5 exons. **(E)** The distribution of the length of the novel *PRMT1* circRNAs. The majority of circRNAs have a length between 200 nt and 600 nt. The moving average trendline is also depicted, in order to smooth out fluctuations in the values. **(F)** Frequency plot of the copy number variation of *PRMT1* in the 11 breast cancer cell lines grouped by disease subtype, according to the DeepMAP portal of the Cancer Cell Line Encyclopedia (CCLE). Out of the 11 cell lines, 4 have a diploid copy number of *PRMT1*, while the other 7 are characterized by 1-3 copy number gains of the gene.

**Supplementary Figure 3.** Illustration of the presence of all identified novel *PRMT1* circRNAs across the 12 cell lines, sorted by frequency. The teal blue color indicates the presence of the respective circRNA in a cell line, according to the results of our targeted nanopore sequencing workflow. For each circRNA, the GenBank^®^ accession number is shown, as well.

**Supplementary Figure 4.** Representation of the 3 novel *PRMT1* exons, in linear and circular form, as well as the circRNAs that contain poly(A) stretches. **(A)** Depiction of the coverage of *PRMT1* gene in the 3 breast cancer cell lines where the novel exons were found (logarithmic scale), as well as the alignment of the circRNAs that contain these novel exons, through the Integrative Genomics Viewer. Notably, novel exon #2 is found to be expressed in both the HCC70 and BT-474 cell lines, while novel exon# 3 is incorporated into 2 circRNAs and is the most frequent novel exon. **(B)** The exonic composition of the *PRMT1* circRNAs that contain the 3 novel exons, shown in circular form (not on scale). These circRNAs vary in their length as well as their structure, and novel exons #1 and #3 participate in the back-slice junction (BSJ). Regarding novel exon #3, it was found with 2 different 3΄ splice sites; in circ-PRMT1-90 it is spliced with the known 5΄ end of exon 12, while in circ-PRMT1-103 it is 3΄ truncated and spliced with a 5 nucleotide 5΄ extension of exon 12. **(C)** The exonic composition of the *PRMT1* circRNAs that incorporate poly(A) stretches, shown in circular form (not on scale). These circRNAs were found in the T-47D and SK-BR-3 cell lines, respectively. In both cases, the poly(A) stretches participate in the back-splice junction (BSJ).
