# Supplementary material for: Targeted nanopore sequencing for the identification of novel PRMT1 circRNAs unveils a diverse transcriptional profile of this gene in breast cancer cells
Source: Genes Dis. 2023 May 18;11(2):589–92. doi: 10.1016/j.gendis.2023.04.013 (PMC10491911; doi:10.1016/j.gendis.2023.04.013)
Supplement: Multimedia component 2 [file mmc2.docx]

**Supplementary materials and methods**

**Cell culture**

In this study, eleven breast cancer cell lines of distinct molecular subtypes (Suppl. Table 1) and a normal epithelial breast cell line (MCF-12A) were used. All cell lines were cultured according to the American Type Culture Collection (ATCC^®^) instructions, at 37 °C and a humidified atmosphere of 5% CO_2_.

**Total RNA isolation and reverse transcription**

Following cell lysis, total RNA extraction from each cell line was performed using the TRItidy G™ Reagent (AppliChem GmbH, Darmstadt, Germany), following the manufacturer’s instructions. All RNA samples were diluted in DEPC-treated H_2_O and stored at -80 °C until further use. A BioSpec-nano Micro-volume UV-Vis Spectrophotometer (Shimadzu, Kyoto, Japan) was used to evaluate the concentration and purity of the RNA extracts. Next, the integrity of the RNA samples was assessed by agarose gel electrophoresis.

Then, reverse transcription was carried out, using 2 μg of each RNA extract as a template to conduct cDNA synthesis. First-strand cDNA was synthesized using M-MLV reverse transcriptase (Life Technologies Ltd., Carlsbad, CA, USA) and 50 ng random hexamers (New England Biolabs Ltd., Hitchin, UK), as per the guidelines provided by the manufacturer. A 20 μL volume of cDNA was produced by this procedure, and all steps were carried out in a MiniAmp Thermal Cycler (Applied Biosystems™, Thermo Fisher Scientific Inc.).

**Primer design and two-round PCR assays**

Two pairs of divergent (outward facing) primers were designed for each of the 12 annotated *PRMT1* exons, in order to specifically amplify cDNAs from the *PRMT1* circRNAs, and not the linear alternative transcripts, as previously described (Suppl. Table 2). This way, there is no need for a circRNA enrichment step in the process, since the amplification of cDNAs derived from linear transcripts is avoided.

The cDNAs that were generated in the previous step were then subjected to first-round PCR assays, using an outer pair of divergent primers for each *PRMT1* exon. The PCR products from these reactions were diluted at a ratio of 1:100 in molecular grade nuclease-free H_2_O and were used as template for nested PCR assays, using the inner pair of divergent primers for each exon. All first and second-round PCR assays were conducted in a MiniAmp Thermal Cycler (Applied Biosystems™), under the following cycling conditions: a denaturation step at 95 ^o^C for 3 min, followed by 25 cycles of 95 ^o^C for 30 sec, an annealing step at the optimal Ta for each primer pair for 30 sec, 72 ^o^C for 1 min, and a final elongation step at 72 ^o^C for 1 min.

Finally, the nested PCR products of each cell line were mixed at equal volumes (10 μL) and purified using spin columns (MACHEREY-NAGEL GmbH & Co. KG, Düren, Germany), in 30μL of Elusion Buffer NE. The concentration of the purified PCR products for each of the 12 cell lines was determined using a Qubit fluorometer (Thermo Fisher Scientific, Inc.).

**DNA library construction and adapter ligation**

DNA libraries were prepared according to the Oxford Nanopore Technologies plc. SQK-LSK109 protocol. More specifically, 86.3 fmol of each purified PCR product mix were used in order to prepare the DNA ends for adapter ligation, using the NEBNext FFPE Repair Mix and NEBNext Ultra II End repair/dA-tailing Module (New England Biolabs Ltd). Then, barcodes were selected to be attached at the ends of each PCR product mix, so that the products deriving from each of the 12 cell lines could be distinguished. For this purpose, the Blunt/TA Ligase Master Mix (New England Biolabs Ltd), the Native Barcoding Expansion kit EXP-NBD114 (Oxford Nanopore Technologies plc.), and the NEBNext Quick Ligation Module (New England Biolabs Ltd) were used.

**Targeted long-read sequencing with nanopore technology**

Prior to library loading, the flow cell was primed using the flow cell priming kit (EXP-FLP002; Oxford Nanopore Technologies plc.), and 18 fmol of DNA library were then used to prepare the Sequencing Mix using the Flongle Sequencing Expansion (EXP-FSE001; Oxford Nanopore Technologies plc.), which was added to the flow cell. Targeted third-generation sequencing with nanopore technology was performed in the MinION Mk1C platform, using the Flongle adapter (FLO-FLG001; Oxford Nanopore Technologies plc.).

**Nanopore sequencing data analysis**

After the completion of sequencing and base calling, raw sequencing data is provided in FAST5 format files. Guppy (v22.05.8) from the MinKNOW software, which is integrated into the operating system of the MinION platform, was used to base call the raw sequencing data, and create FASTQ files. To process the data, both publicly available algorithms and algorithms designed by our research group specifically for the identification of circRNAs were used. Thus, the FASTQ files containing the sequences of the “passed” reads, excluding the “failed” ones, were aligned against chromosome 19 (version NC_000019.10) using the Minimap2 algorithm.^1^ SAMtools and BEDtools were then used to generate SAM, BAM, and BED files, containing the sequence alignment information obtained from the experiment.^2, 3^

Sensitive custom algorithms designed in PERL programming language were used to identify new circRNAs of the *PRMT1* gene. In particular, the ASDT algorithm^4^ was used to detect the back-splicing events when a modified *PRMT1* GenBank^®^ file is provided as input. Then, the ASDT remodeler (<https://github.com/pkarousi/ASDT_remodeler>) and the Read catcher (<https://github.com/pkarousi/Read_catcher>) algorithms were used to provide the reads corresponding to circRNAs. The selected reads representing circRNAs were then manually annotated. The Integrative Genomics Viewer (IGV)^5^ was also used for the visualization of the BAM files from each cell line, so that novel exons and novel splice sites of *PRMT1* circRNAs, in general, were detected. Moreover, copy number variation (CNV) analysis was performed for *PRMT1* in the 11 breast cancer cell lines through the Cancer Cell Line Encyclopedia, in order to assess any correlation with the number of identified *PRMT1* circRNAs. Finally, the miRNA binding sites of the circRNAs incorporating novel *PRMT1* genomic regions were predicted using the miRDB database's custom prediction tool^6^, and the ORF Finder bioinformatics tool (<https://www.bioinformatics.org/sms2>) was used for the query of open reading frames in the *PRMT1* circRNA sequences.

**References**

1. Li H. Minimap2: pairwise alignment for nucleotide sequences. *Bioinformatics*. 2018;34(18):3094-3100.

2. Danecek P, Bonfield JK, Liddle J, et al. Twelve years of SAMtools and BCFtools. *Gigascience*. 2021;10(2)

3. Quinlan AR, Hall IM. BEDTools: a flexible suite of utilities for comparing genomic features. *Bioinformatics*. 2010;26(6):841-2.

4. Adamopoulos PG, Theodoropoulou MC, Scorilas A. Alternative Splicing Detection Tool-a novel PERL algorithm for sensitive detection of splicing events, based on next-generation sequencing data analysis. *Ann Transl Med*. 2018;6(12):244.

5. Robinson JT, Thorvaldsdottir H, Winckler W, et al. Integrative genomics viewer. *Nat Biotechnol*. 2011;29(1):24-6.

6. Chen Y, Wang X. miRDB: an online database for prediction of functional microRNA targets. *Nucleic Acids Res*. 2020;48(D1):D127-D131.
