# Supplementary material for: Targeted nanopore sequencing for the identification of novel PRMT1 circRNAs unveils a diverse transcriptional profile of this gene in breast cancer cells
Source: Genes Dis. 2023 May 18;11(2):589–92. doi: 10.1016/j.gendis.2023.04.013 (PMC10491911; doi:10.1016/j.gendis.2023.04.013)
Supplement: Multimedia component 3 [file mmc3.docx]

**Suppl. Table 1.** Characteristics of the 11 human breast cancer cell lines that were used in this study.

| **Cell line** | **Type** | **ER^a^ status** | **PR^b^ status** | **HER2^c^ status** | **Molecular subtype** |
| --- | --- | --- | --- | --- | --- |
| BT-20 | Carcinoma | **-** | **-** | **-** | Triple-negative |
| MDA-MB-231 | Adenocarcinoma | **-** | **-** | **-** | Triple-negative |
| MDA-MB-468 | Adenocarcinoma | **-** | **-** | **-** | Triple-negative |
| Hs578T | Carcinoma | **-** | **-** | **-** | Triple-negative |
| MDA-MB-453 | Metastatic carcinoma | **-** | **-** | **-** | Triple-negative |
| HCC70 | Ductal carcinoma | **-** | **-** | **-** | Triple-negative |
| MCF-7 | Adenocarcinoma | **+** | **+** | **-** | Luminal A |
| ZR-75-1 | Ductal carcinoma | **+** | **+** | **-** | Luminal A |
| T-47D | Ductal carcinoma | **+** | **+** | **-** | Luminal A |
| BT-474 | Ductal carcinoma | **+** | **+** | **+** | Luminal B |
| SK-BR-3 | Adenocarcinoma | **-** | **-** | **+** | HER2-positive |

^a^ estrogen receptor; ^b^ progesterone receptor; ^c^ human epidermal growth factor receptor 2.
