# Supplementary material for: Targeted nanopore sequencing for the identification of novel PRMT1 circRNAs unveils a diverse transcriptional profile of this gene in breast cancer cells
Source: Genes Dis. 2023 May 18;11(2):589–92. doi: 10.1016/j.gendis.2023.04.013 (PMC10491911; doi:10.1016/j.gendis.2023.04.013)
Supplement: Multimedia component 4 [file mmc4.docx]

**Suppl. Table 2.** The pairs of divergent primers used in the first and nested PCRs to amplify cDNAs deriving from *PRMT1* circRNAs.

| **Annealing site** | **First PCR** | | | **Nested PCR** | | |
| --- | --- | --- | --- | --- | --- | --- |
|  | **Primer direction** | **Primer sequence (5΄🡪3΄)** | **Amplicon T_a_ (^o^C)** | **Primer direction** | **Sequence (5΄🡪3΄)** | **Amplicon T_a_ (^o^C)** |
| Exon 1 | Sense | AGGCCGCGAACTGCATC | 63 | Sense | CGCGAACTGCATCATGGAG | 60 |
|  | Antisense | GGCTGCCGCCATCTTCA |  | Antisense | GCCGCCATCTTCACCC |  |
| Exon 2 | Sense | GGATGAGCCTCCAGCCG | 62 | Sense | CCAGCCGCCTCTTGAA | 58 |
|  | Antisense | GCTCATCCCATTAGCCAAGGT |  | Antisense | AGCCAAGGTGGCTACAAAATT |  |
| Exon 3 | Sense | AGCTAGAGACGGGGTCAGAG | 62 | Sense | GAGAGATGGTAGGCGTGGC | 61 |
|  | Antisense | GGGGCAGACACAGGGAA |  | Antisense | AGACACAGGGAAGGGCAAAG |  |
| Exon 4 | Sense | TGACTCCTACGCACACTTTGG | 60 | Sense | CACACTTTGGCATCCACGAG | 60 |
|  | Antisense | TCTTTGGATGTCATGTCCTCAGC |  | Antisense | TTGGGCTTCTCACTGCTTTCC |  |
| Exon 5 | Sense | CGGCACCTCTTCAAGGACAA | 60 | Sense | GGCATCCTCTGCATGTTTGC | 60 |
|  | Antisense | ATGAAACATGGAGTTGCGGT |  | Antisense | CCTCGTCCTTCAGCATCTC |  |
| Exon 6 | Sense | CGGTGAAGATCGTCAAAGC | 57 | Sense | AGCCAACAAGTTAGACCACG | 58 |
|  | Antisense | AATCAGAGATACTGGAACACTCGAT |  | Antisense | AATCAGAGATACTGGAACACTCGAT |  |
| Exon 7 | Sense | GAGTCCATGCTCAACACCGT | 61 | Sense | CTATGCCCGGGACAAGTGG | 61 |
|  | Antisense | GAAGAGGCAGTAGCCCATCC |  | Antisense | TCGCTGATGATGATGTCCACC |  |
| Exon 8 | Sense | TGTGACGGCCATCGAGGA | 63 | Sense | GGACCGGCAGTACAAAGACT | 60 |
|  | Antisense | GCCCGGTCTGGGAAGATG |  | Antisense | GATGAGGCCATCGGGC |  |
| Exon 9 | Sense | GATGTCGTGGACCCCAAACA | 61 | Sense | ACCAACGCCTGCCTCATAAA | 60 |
|  | Antisense | GGCTCCTTAATGGCCACATCT |  | Antisense | AGCCATACACGTTCTCCCAC |  |
| Exon 10 | Sense | CCCTGGTGGCCTACTTCAAC | 61 | Sense | TTCACACGCTGCCACAAGA | 60 |
|  | Antisense | GCTTCACTTGCAGGCAGAAC |  | Antisense | TCAGGTCTTCCACCTTGACG |  |
| Exon 11 | Sense | TACCTGACCGTGAAGACGG | 60 | Sense | GAGATCTTCGGCACCATCGG | 61 |
|  | Antisense | TCCATGTAGAACACCGTCTGC |  | Antisense | GTCTGCTTCCAGTGCGTGTA |  |
| Exon 12 | Sense | CTGTCCTGCTCCACCGACTA | 61 | Sense | AGCGTTCCTAGGCGGTTTC | 61 |
|  | Antisense | ACAGCTGGCCCTTGAAGTC |  | Antisense | CGATGGTGAAGTCCAGGTCC |  |
