# Supplementary material for: Targeted nanopore sequencing for the identification of novel PRMT1 circRNAs unveils a diverse transcriptional profile of this gene in breast cancer cells
Source: Genes Dis. 2023 May 18;11(2):589–92. doi: 10.1016/j.gendis.2023.04.013 (PMC10491911; doi:10.1016/j.gendis.2023.04.013)
Supplement: Multimedia component 5 [file mmc5.docx]

**Suppl. Table 3.** Attributes of the obtained nanopore sequencing reads and the identified *PRMT1* circRNAs resulting from the bioinformatic analysis of this targeted sequencing data.

|  | **BT-20** | **MDA-ΜΒ-231** | **MDA-ΜΒ-468** | **Hs578T** | **MDA-ΜΒ-453** | **HCC70** | **MCF-7** | **ZR-75-1** | **T-47D** | **BT-474** | **SK-BR-3** | **MCF-12A** |
| --- | --- | --- | --- | --- | --- | --- | --- | --- | --- | --- | --- | --- |
| **Maximum read length (nt**^a^**)** | 1,573 | 2,551 | 1,764 | 2,377 | 1,931 | 5,810 | 4,087 | 2,083 | 2,370 | 3,488 | 3,318 | 2,536 |
| **Minimum read length (nt**^a^**)** | 131 | 138 | 131 | 111 | 131 | 111 | 141 | 143 | 154 | 140 | 121 | 118 |
| **Mean read length (nt**^a^**)** | 393 | 386 | 381 | 393 | 388 | 427 | 392 | 444 | 415 | 411 | 403 | 441 |
| **Identified circRNAs** | 60 | 40 | 44 | 61 | 58 | 74 | 47 | 49 | 54 | 70 | 74 | 58 |
| **Maximum circRNA length (nt**^a^**)** | 1,501 | 639 | 1,260 | 1,501 | 1,501 | 1,193 | 802 | 1,501 | 804 | 804 | 1,501 | 1,627 |
| **Minimum circRNA length (nt**^a^**)** | 165 | 165 | 165 | 153 | 153 | 153 | 153 | 153 | 165 | 153 | 153 | 165 |
| **Mean circRNA length (nt**^a^**)** | 411 | 366 | 401 | 362 | 371 | 387 | 365 | 414 | 403 | 359 | 404 | 449 |

^a^ Nucleotides.
