# Supplementary material for: Targeted nanopore sequencing for the identification of novel PRMT1 circRNAs unveils a diverse transcriptional profile of this gene in breast cancer cells
Source: Genes Dis. 2023 May 18;11(2):589–92. doi: 10.1016/j.gendis.2023.04.013 (PMC10491911; doi:10.1016/j.gendis.2023.04.013)
Supplement: Multimedia component 6 [file mmc6.docx]

**Suppl. Table 4.** The novel *PRMT1* circRNAs that are exclusively present either in one molecular subtype of breast cancer or in the normal breast cancer cell line.

| **Molecular subtype of breast cancer** | | | | **Normal breast cells** |
| --- | --- | --- | --- | --- |
| **Triple-negative** | **Luminal A** | **Luminal B** | **HER2-positive** |  |
| circ-PRMT1-18 | circ-PRMT1-16 | circ-PRMT1-87a | circ-PRMT1-61 | circ-PRMT1-34 |
| circ-PRMT1-26 | circ-PRMT1-19 | circ-PRMT1-90 | circ-PRMT1-94 | circ-PRMT1-37 |
| circ-PRMT1-31 | circ-PRMT1-21 | circ-PRMT1-98 | circ-PRMT1-96 | circ-PRMT1-39 |
| circ-PRMT1-47 | circ-PRMT1-23 | circ-PRMT1-103 | circ-PRMT1-99 | circ-PRMT1-43 |
| circ-PRMT1-55 | circ-PRMT1-25 |  | circ-PRMT1-107 | circ-PRMT1-44 |
| circ-PRMT1-66 | circ-PRMT1-59 |  | circ-PRMT1-112 | circ-PRMT1-117 |
| circ-PRMT1-73 | circ-PRMT1-64 |  | circ-PRMT1-114 |  |
| circ-PRMT1-76 | circ-PRMT1-93 |  |  |  |
| circ-PRMT1-78 | circ-PRMT1-95 |  |  |  |
| circ-PRMT1-86 |  |  |  |  |
| circ-PRMT1-108 |  |  |  |  |
| circ-PRMT1-118 |  |  |  |  |
